# Analysis of a conditional gene trap reveals that *tbx5a* is required for heart regeneration in zebrafish

**DOI:** 10.1371/journal.pone.0197293

**Published:** 2018-06-22

**Authors:** Viktorija Grajevskaja, Diana Camerota, Gianfranco Bellipanni, Jorune Balciuniene, Darius Balciunas

**Affiliations:** 1 Department of Biology, College of Science and Technology, Temple University, Philadelphia, PA, United States of America; 2 Department of Zoology, Faculty of Natural Sciences, Vilnius University, Vilnius, Lithuania; Institut Curie, FRANCE

## Abstract

The ability to conditionally inactivate genes is instrumental for fine genetic analysis of all biological processes, but is especially important for studies of biological events, such as regeneration, which occur late in ontogenesis or in adult life. We have constructed and tested a fully conditional gene trap vector, and used it to inactivate *tbx5a* in the cardiomyocytes of larval and adult zebrafish. We observe that loss of *tbx5a* function significantly impairs the ability of zebrafish hearts to regenerate after ventricular resection, indicating that Tbx5a plays an essential role in the transcriptional program of heart regeneration.

## Introduction

Conditional induction of loss-of-function mutations using the Cre-lox system has enabled thorough mechanistic studies of all biological processes, from development to organ homeostasis and behavior, in the mouse model system [1,2,3,4]. Inability to reliably insert loxP sites into desired locations in the genome, despite recent progress [5,6], has hampered conditional loss-of-function studies in zebrafish. Instead, scientists have to rely on inducible overexpression of active and dominant negative proteins to study development and regeneration [7,8,9].

Organisms vary greatly in their regenerative capacity. Among vertebrates, many anamniotes, including the axolotl and the zebrafish, can regenerate a variety of organs, tissues and cell types (reviewed in[10]). Humans are on the other end of the regenerative spectrum, as injury typically results in scar formation. Until recently it was thought that laboratory mice also have limited regenerative capacity. Two recent findings challenge that notion: observation that newborn mice can heal ventricular injury [11], and that a related mouse species, the African spiny mouse, can regenerate skin lesions [12]. These observations suggest that with detailed mechanistic knowledge of the process, it may be possible to re-activate dormant regenerative programs in other mammals as well.

Signaling through classical developmental pathways including Wnt, Sonic Hedgehog, BMP, Retinoic Acid, and TGF is essential during regeneration ([10,13,14,15] and references therein). These observations support the notion of a significant overlap between genetic mechanisms governing development and regeneration, necessitating the use of conditional mutants to study regeneration. However, conditional loss of function mutants can only be robustly generated in the laboratory mouse, which has poor regenerative capacity. Conversely, in vertebrate model systems with extensive regenerative capacity such as the salamander or the zebrafish only inducible dominant negative approaches in conjunction with small molecule exposure and morpholino knockdowns have been used to study regeneration [15].

In this report, we have constructed and tested fully conditional, highly mutagenic gene trap vectors. They combine high mutagenicity of GBT vectors [16,17,18,19] with the ability to conditionally revert and re-induce gene inactivation by turning the gene trap cassette around. In further contrast to other conditional gene trap vectors [20,21,22], we employ simple +10/-10 mutant loxP and FRT sites instead of using the more cumbersome FLEx switch (reviewed in [23]). Using *tbx5a*^*tpl58*^ as the model gene trap locus, we demonstrate that these +10/-10 loxP and FRT sites can be readily used to stably invert the gene trap cassette in larval and adult zebrafish, and observe that *tbx5a* is required for cardiac regeneration.

## Results and discussion

To facilitate genetic analysis of pleiotropic genes and biological processes which occur late in ontogenesis, we have developed a highly mutagenic and fully conditional 5’ gene trap with Gal4-VP16 as the primary gene trap reporter. Our approach is similar to the one employed by the mouse gene trap consortium [24] and previous reports in zebrafish [21,25] with two key differences. First, while previous reports used the FLEx switch based on linker mutant site-specific recombinase (SSR) sites, we took advantage of the much more compact nature of LE/RE mutant SSR sites to achieve the same goal. Second, we used components which have been previously validated to be very effective at inducing null mutations upon integration into introns of genes [16,17,18,19,26,27].

**Fig 1:**
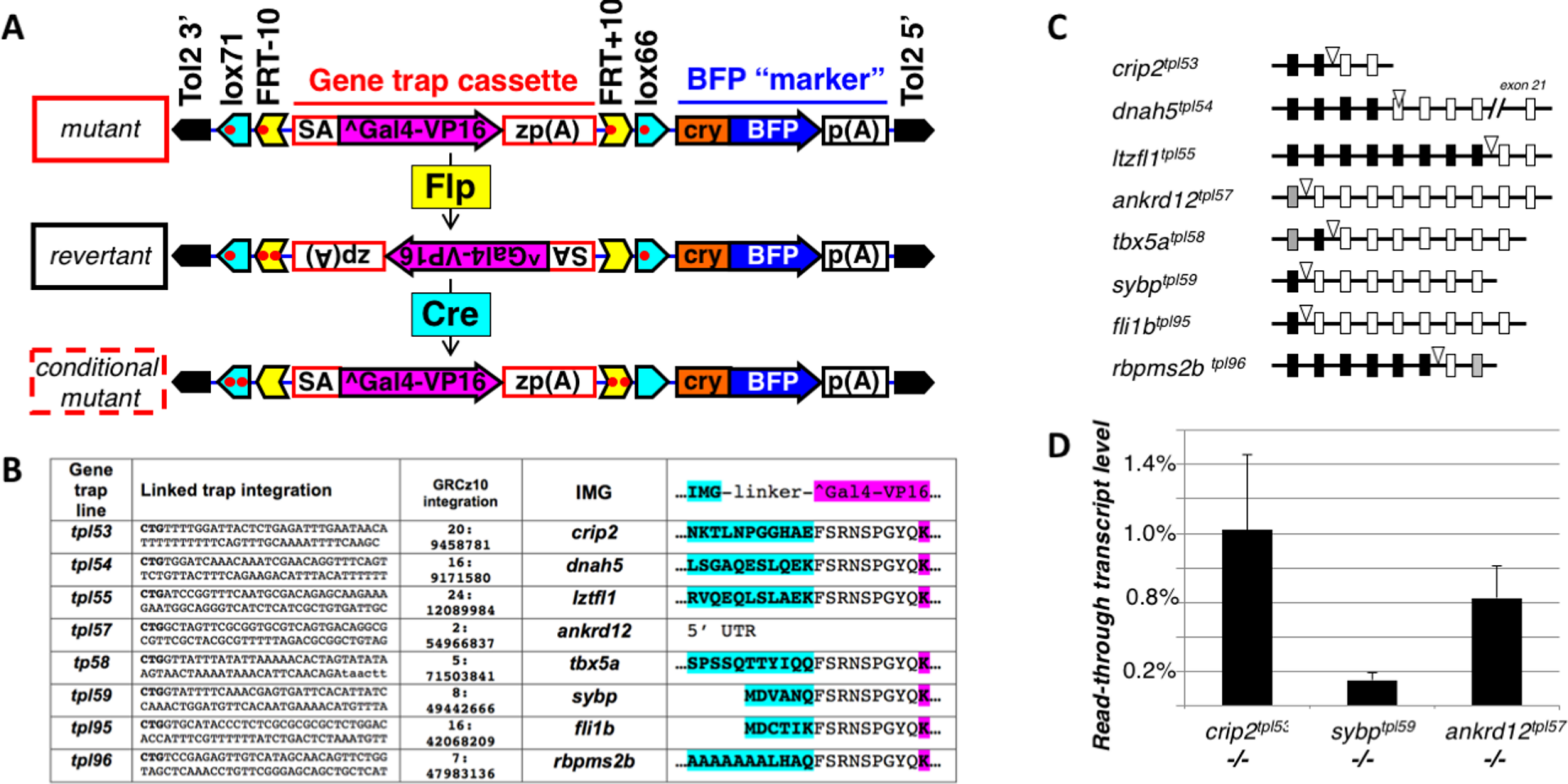
Tol2-based On/Off/On (“Switchblade”, GBT-S1/S8) vectors for insertional mutagenesis and characterized gene trap lines. **A.** Diagram of the vector and conditional regulation. The gene trap cassette is flanked by lox71, FRT-10, FRT+10 and lox66 sites. Components responsible for high degree of mutagenicity are shown in red. SA, carp beta actin splice acceptor, ^Gal-VP16, AUG-less Gal4-VP16, zp(A), zebrafish beta actin 3’ UTR and transcriptional termination sequences, cry, *X*. *laevis* gamma crystalline promoter, p(A), SV40 poly(A). The gene trap cassette is identical to that used in GBT-B1 gene trap vector (Balciuniene et al., 2013). Expression Flp recombinase will result in inversion of the gene trap cassette and one wild type (FRT) and one double mutant, inactive (FRT+10/-10, two red dots) site. Expression of the Cre recombinase will result in second inversion of the cassette conditionally mutating the gene. **B.** GBT-S1 and GBT-S8 gene trap lines. First column, gene trap line. Second column, sequence adjacent to the 3’ end of the gene trap integration. The capital CTG are the last three nucleotides of Tol2. Third column, location of the gene trap integration on the GRCz10 zebrafish genome assembly. Fourth column, insertionally mutated gene (IMG). Fifth column, sequence of the IMG-Gal4-VP16 fusion protein, IMG sequence is highlighted in aqua, Gal4 sequence is highlighted in magenta. The “linker” sequence is encoded by the linker between splice acceptor and Gal4 in GBT-S1 and GBT-S8. **C.** Diagram of gene trap loci. Gene trap integration site is shown as a triangle, with mutated gene’s exons depicted as squares: grey for non-coding, black for 5’ of integration site and white for 3’ of integration site. **D.** Assessment of transcript levels by quantitative RT-PCR. Three non-phenotypic lines were chosen for analysis. Quantitative RT PCR was performed on pools of RNA from wild type and homozygous mutant 5 dpf embryos, and normalized to beta actin. Error bars represent standard deviation.

We constructed a miniTol2 vector, GBT-S1, with the Gal4-VP16 gene trap cassette flanked by LE/RE modified loxP (lox66, lox72) and FRT (FRT+10, FRT-10) SSR sites **(Fig 1A)**. Since overexpression of Gal4-VP16 has been shown to lead to toxicity [28,29], we considered using a less transcriptionally potent derivative Gal4-FF. We tested Gal4-FF [28] in the context of GBT-B1 gene trap vector [19] but failed to recover any gene trap lines (Balciunas et al., unpublished). Even though this negative observation is insufficient to draw firm conclusions, we implied that Gal4-FF may be insufficiently potent to function in a highly stringent gene trap and elected to continue using Gal4-VP16. A lens-specific BFP expression cassette was added to expedite identification of embryos which inherit the gene trap. Plasmid containing the gene trap (25 pg) was injected along with 25 pg of Tol2 transposase mRNA into the yolks of zebrafish embryos at 1-cell stage. Lens BFP-positive embryos were raised and screened by crossing to the Tg(*UAS*:*mRFP*)*tpl2* reporter line [19,30].

Out of 115 potential F0 fish screened, twelve produced RFP-positive progeny. We focused on seven lines with the most distinct mRFP expression patterns and identified insertionally mutated genes in six of them: *crip2*, *dnah5*, *lztfl1*, *ankrd12*, *tbx5a* and *sybp*. We observed that in many gene trap lines, BFP expression was too weak to reliably identify trap-positive fish, and therefore constructed a second vector, GBT-S8, with a longer gamma-crystalline promoter driving BFP, and with the marker cassette cloned in reverse orientation. A small screen focused on the cardiovascular system produced two additional gene trap lines, *rbpms2b* and *fli1b* (**Fig 1B and 1C**).

Incrosses of fish heterozygous for six gene traps (*crip2*, *dnahc5*, *lztfl1*, *ankrd12*, *sybp* and *fli1b*) resulted in phenotypically normal embryos. Crossing fish heterozygous for *rbpms2b* gene trap resulted in approximately 25% of progeny developing pericardial edema at 3 days post fertilization (data not shown). We performed quantitative RT-PCR to assess levels of read-through transcripts *crip2*, *ankrd12* and *sybp* homozygotes (**Fig 1D**). Transcript levels in all three lines were below 1%, indicating that mutagenicity of pGBT-S1, similarly to that of parental vector pGBT-B1 [19], is close to that of the extremely mutagenic GBT-RP2 [18] and higher than that of other previously published conditional gene traps [21,25].

**Fig 2:**
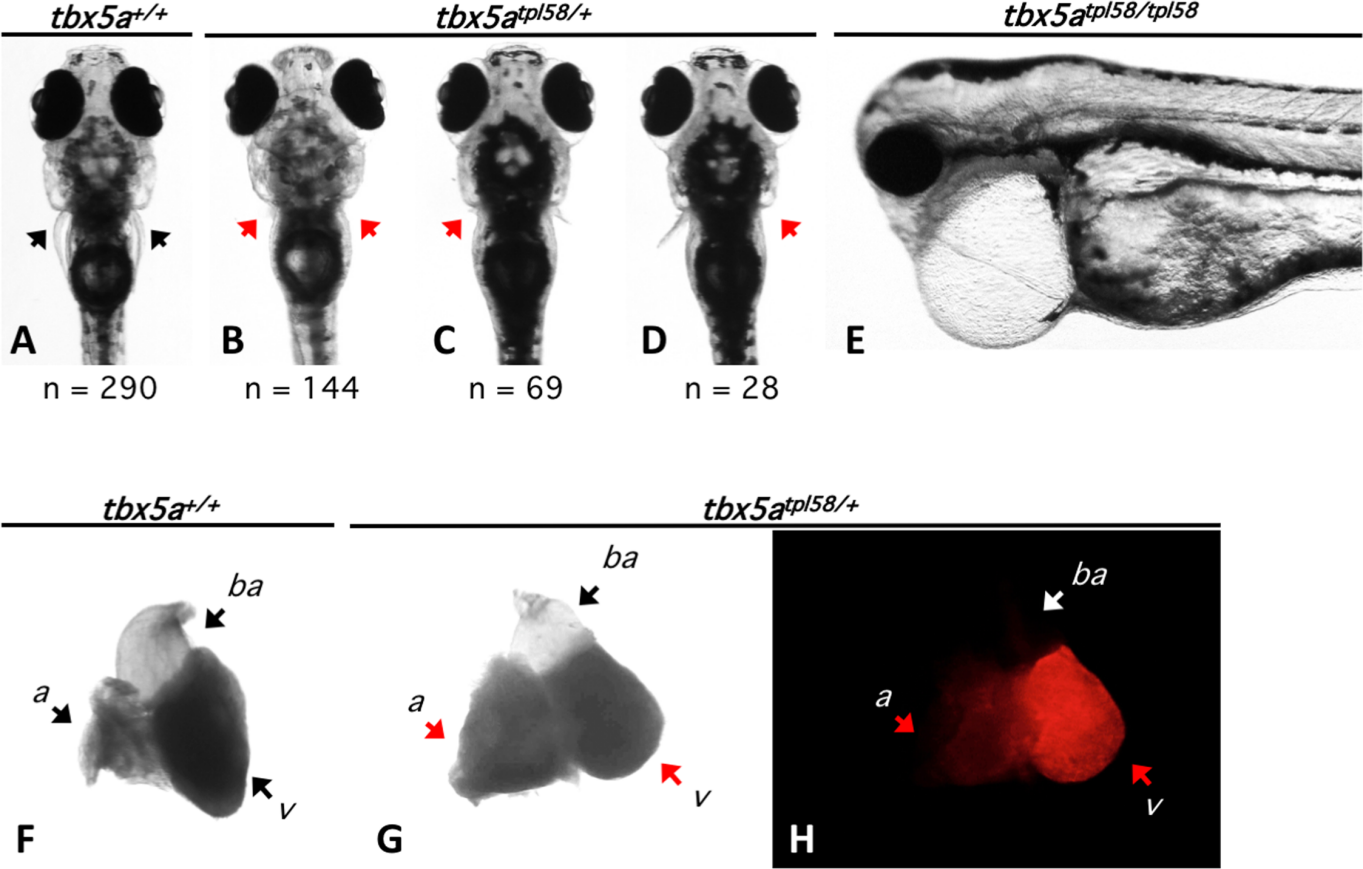
***tbx5a*^*tpl58*^ mutant phenotypes. A-D.** Larvae heterozygous for *tbx5a*^*tpl58*^ gene trap allele display severe but variable fin defects at 5 days post fertilization. Compared to wild type siblings (a), heterozygous mutants display bilaterally equal fin truncation (b), or unequal fin truncation with left (c) or right (d) pectoral fin more severely affected. **E.** Gene trap homozygotes display a phenotype similar to the more severe version of *heartstrings* phenotype described previously (Fig 1D and 1E in Garrity *et al*., 2002). **F-H.** All adults heterozygous for the gene trap had hearts with enlarged atria and mis-shapen (blunt) ventricles (n = 13). *a*, atrium, *v*, ventricle, *ba*, *bulbus arteriosus*.

We chose to focus our attention on the *tbx5a*^*tpl58*^ gene trap line. Tbx5 is a highly conserved transcription factor known to be required for heart and upper limb development in different vertebrate species, and TBX5 haploinsufficiency causes Holt-Oram syndrome in humans [31,32,33,34,35]. Accordingly, we noted that embryos heterozygous for *tbx5a*^*tpl58*^ have abnormal pectoral fins (n = 241) (**Fig 2**). The majority (60%) of examined *tbx5a*^*tpl58/+*^ embryos had equally affected pectoral fins. One third of the *tbx5a*^*tpl58/+*^ (29%) had the left pectoral fin more affected than a right. Only 11% of the *tbx5a*^*tpl58/+*^ showed more affected right pectoral fin. We noticed that similarly to observations in human patients with Holt-Oram syndrome, the left upper limb was more likely to be more severely affected than the right (compare **Figs 2A–2D** to 1 in [36]). We observed poor and highly variable survival of *tbx5a*^*tpl58/+*^ embryos to adulthood. All examined adults (n = 48) lacked pectoral fins. We also noted that all hearts dissected from *tbx5a*^*tpl58/+*^ fish (n = 13) had enlarged atria and bluntly shaped ventricles (**Fig 2G and 2H**), similar to the hearts of mice heterozygous for *tbx5* deletion [33]. Crosses of fin-less males and females heterozygous for *tbx5a*^*tpl58*^ failed to produce embryos. We therefore used the sperm of *tbx5a*^*tpl58/+*^ males to *in vitro* fertilize the eggs of *tbx5a*^*tpl58/+*^ females, and observed 25% of embryos with linear heart phenotype (**Fig 2E,** n = 7 genotyped). This phenotype closely resembles the more severe version of the *heartstrings* phenotype caused by the *hst* allele of *tbx5a* [35]. Notably, our gene trap truncates the Tbx5a protein after the first 50 amino acids while *tbx5a*^*hst*^ introduces a stop codon in the second to last exon, at the amino acid 316, after the T-box DNA binding domain. The observations that amino acids 1–239 are sufficient to bind DNA and form a structural dimer with Nkx2.5 [37], suggest that the protein encoded by *tbx5a*^*hst*^ may be a hypomorph. Other explanations such as alternative splicing or nonsense read-through of *tbx5a*^*hst*^ are possible as well [38,39]. A particularly intriguing possibility would be that point mutant *tbx5a*^*hst*^ but not gene trap mutant *tbx5a*^*tpl58*^ may induce genetic compensation [40,41]. Alternatively, the more severe phenotypes of *tbx5a*^*tpl58*^, despite striking similarity to the mouse and human Tbx5 haploinsufficiency phenotypes, could be attributed to toxicity of Gal4-VP16 [28,29].

Tbx5 is an essential component of transcription factor cocktails capable of trans-differentiating fibroblasts into cardiomyocytes [42,43,44]. Together with the fact that Tbx5 is essential for heart development, this suggests that Tbx5 may be required for cardiac regeneration. On the other hand, *de novo* differentiation of cardiomyocytes from stem cells does not appear to play a major role in cardiac regeneration in zebrafish or neonatal mice [9,11,45]. It is also not clear if *tbx5a* is upregulated in response to cardiac injury in adult zebrafish [7,45,46], although it is induced in injured embryonic hearts [47]. We therefore decided to use our gene trap mutant to test if *tbx5a* may be required for cardiac regeneration in adult zebrafish.

We first tested if fish heterozygous for the *tbx5a*^*tpl58*^ gene trap are able to regenerate their hearts after ventricular resection. We found that while the hearts of wild type fish were completely healed 30 days after ventricular resection (n = 6), the hearts of *tbx5a*^*tpl58*^ heterozygotes retained a significant amount of scar tissue (n = 3) (data not shown), which indicates a regeneration defect. However, the observed regeneration defect may be an indirect consequence of morphological abnormalities described above (**Fig 2G and 2H**).

To determine if *tbx5a* is directly involved in regeneration we took advantage of the conditional inversion components built into our gene trap vector. We first tested if inversion of the gene trap cassette using Flp recombinase will result in abrogation of all mutant phenotypes. Embryos obtained from a cross between *tbx5a*^*tpl58*^ heterozygotes and *(UAS*:*mRFP)tpl2* homozygotes and were injected with *in vitro* transcribed Flp^o^ recombinase mRNA. Embryos with reduced mRFP expression were selected and raised to adulthood. One of the adult fish was outcrossed and in the first clutch produced three RFP-positive and 13 RFP-negative embryos. We analyzed all 16 embryos by PCR and confirmed that all three RFP-positive embryos have non-inverted gene trap, and of the 13 RFP-negative embryos, three were positive for the inverted gene trap (**S1 Fig**). Siblings of the tested embryos were raised to adulthood and the reverted gene trap line *tbx5a*^*tpl58R*^ was established. Presence of inverted gene trap was confirmed by PCR and sequencing (**S2 Fig**). Most importantly, *tbx5a*^*tpl58R*^/*tbx5a*^*tpl58R*^ homozygotes are viable and do not display any overt phenotypes. Thus, presence of the inverted gene trap cassette and gamma-crystalline-BFP expression cassette in the intron does not appear to significantly negatively impact the expression of *tbx5a*.

Two experiments were performed to test if the gene trap cassette flanked by LE/RE mutant loxP sites can be inverted again to re-trap the gene in larval zebrafish. First, *tbx5a*^*tpl58R/+*^*; UAS*:*mRFP* heterozygotes were outcrossed and embryos were injected with Cre mRNA, resulting in mosaic mRFP expression in the hearts, pectoral fins and dorsal retina (data not shown). Second, to ascertain feasibility of conditional re-trapping, *tbx5a*^*tpl58R/+*^*; UAS*:*mRFP* heterozygotes were crossed to a line expressing the tamoxifen-inducible Cre under the control of a ubiquitous promoter, Tg*(-3*.*5ubb*:*CreERT2*, *myl7*:*EGFP)cz1702* transgenic line [48]. Two-day old embryos were incubated in embryo water containing 0.5 μM 4-hydroxytamoxifen (4-HT hereafter) for 24 hours as described previously [48,49]. Embryos were scored for mosaic mRFP expression, and DNA was prepared from 5 dpf RFP-positive embryos. Inversion of the gene trap cassette was confirmed by sequencing of PCR fragments (**S2 Fig**).

**Fig 3:**
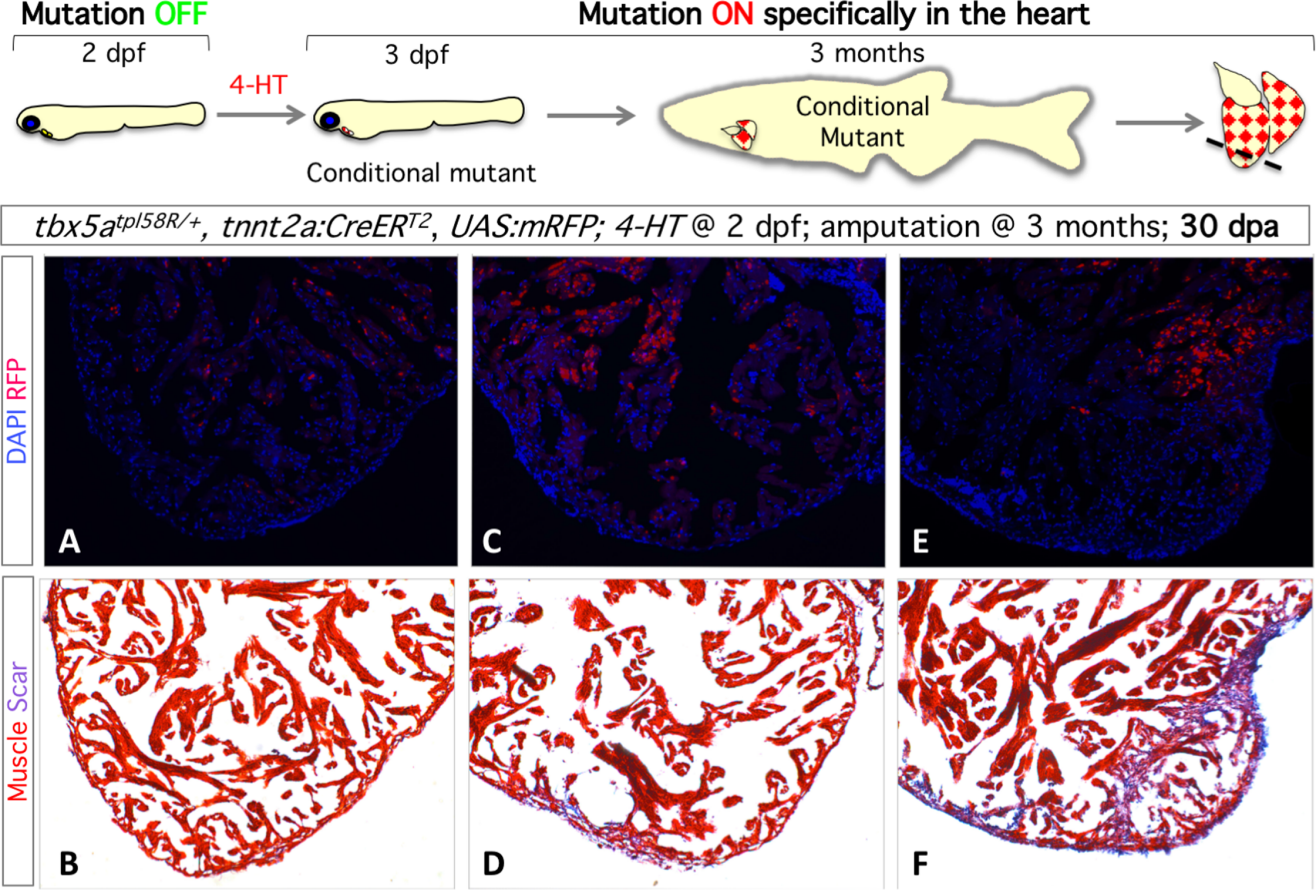
Cardiomyocytes heterozygous for *tbx5a*^*tpl58*^ contribute to the adult heart but lead to impaired regeneration. Top, experimental outline. **A-F.** *Representative* images of adjacent sections (A and B, C and D, E and F) of hearts (n = 7) stained for DAPI (A, C, E) and by Pico-Mallory stain (B, D, F).

For cardiomyocyte-specific expression of CreERT2, we established a Tg*(-3*.*6tnnt2*:*CreERT2*, *gcrygc*:*RFP)tpl48* (*tnnt2*:*CreERT2* henceforth) transgenic line and demonstrated that Cre activity in this line is both tamoxifen-inducible and heart-specific (**S3 Fig**). We generated fish heterozygous for *tnnt2*:*CreERT2*, *UAS*:*mRFP* and *tbx5a*^*tpl58R*^. Embryos were exposed to 4-hydroxytamoxifen (4-HT) for 24 hours starting at 2 days post-fertilization. Mosaic heart-specific mRFP expression (from Tg*(UAS*:*mRFP)tpl2* reporter transgene) was observed in the hearts of 3 dpf embryos, indicating successful inversion of the *tbx5a* gene trap into its mutant form (**S3 Fig**). Embryos exposed to 4-HT and vehicle-exposed controls were raised to adulthood. Three adult fish were sacrificed to test if cardiomyocytes made heterozygous for the re-trapped gene trap (*tbx5a*^*tpl58RT*^) at 2–3 dpf are able to successfully contribute to the adult myocardium using fluorescence from *UAS*:*mRFP* as the readout. Examined hearts had variable numbers of RFP-positive cells, indicating that heterozygous cells indeed are able to contribute to the adult heart. Importantly, all examined hearts were morphologically normal (**S3 Fig**). We then performed ventricular resection on siblings of these mosaic fish (n = 7). One month after injury, only 2/7 examined hearts appeared to have undergone normal regeneration while 3/7 hearts and 2/7 hearts had medium and large amount of collagen deposition, respectively. Adjacent sections were stained for DAPI and visualized for RFP fluorescence. We noted very few RFP-positive cells (and thus heterozygous for *tbx5a*^*tpl58RM*^) in the regenerated myocardium (tip of the ventricle). Further experiments are needed to determine if expression *tbx5a* is switched off in the regenerated myocardium, or if cardiomyocytes with reduced *tbx5a* dosage are less capable of contributing to the regenerating myocardium (**Fig 3**).

Although the experiment described above did indicate that *tbx5a* is likely to play a direct role in cardiac regeneration, we could not exclude the possibility that there are subtle developmental, physiological or cellular defects in hearts mosaic for *tbx5a*^*tpl58RT*^ mutation starting during development. Also, since Tbx5a is required for heart development, it was not feasible to generate homozygous mutant cells and test the ability of hearts containing such cells to regenerate.

**Fig 4:**
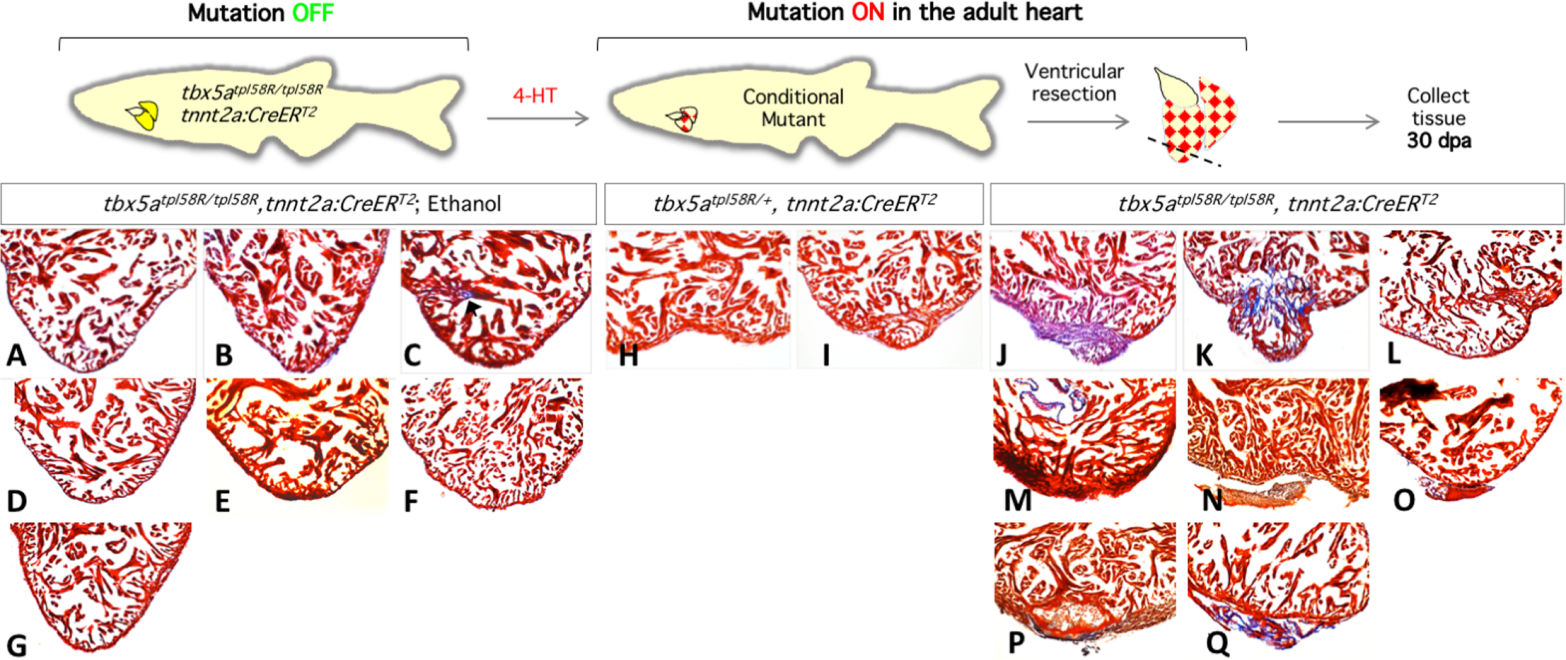
Loss of *tbx5a* in adult zebrafish leads to severe regeneration defects. Top, experimental outline. Bottom, sections of zebrafish hearts stained by Picro-Mallory stain. **A-G.** Hearts of zebrafish (n = 7) homozygous for the reverted *tbx5a* allele, and heterozygous for *tnnt2*:*CreERT2*, treated with ethanol (solvent for 4-HT). **H, I.** Hearts of zebrafish (n = 2) heterozygous for the reverted *tbx5a* allele, and heterozygous for *tnnt2*:*CreERT2*, treated with 4-HT. **J-Q.** Hearts of zebrafish (n = 8) homozygous for the reverted *tbx5a* allele, and heterozygous for *tnnt2*:*CreERT2*, treated with 4-HT.

To overcome these limitations, loss of function mutation had to be induced in the hearts of adult zebrafish. To achieve this goal, we generated adult fish homozygous or heterozygous for *tbx5a*^*tpl58R*^ in heterozygous *tnnt2*:*CreERT2*, *UAS*:*mRFP* background. To re-trap the *tbx5a* locus, 2.5 month old fish were incubated in 5 μM 4-HT three times for 24 h with a one-day resting period between each incubation (**Fig 4**). We then performed ventricular resection. As expected, control fish incubated in vehicle displayed normal regeneration (n = 7), with only one heart showing mild scarring (**Fig 4A–4G**). In contrast, *tbx5a*^*tpl58R/+*^ incubated in 4-HT displayed mild regeneration defects (n = 2) (**Fig 4H and 4I**), while all *tbx5a*^*tpl58****R***/*tpl58****R***^ fish failed to regenerate their hearts (n = 8) (**Fig 4J–4Q**). Apart from observing induction of mRFP fluorescence in whole hearts (data not shown), we did not assess the efficiency of re-trapping. It therefore remains to be determined if variable severity of the regeneration defect (compare **Fig 4J, 4K and 4Q**) can be attributed to different efficiencies of re-trapping, physical proximity of re-trapped cardiomyocytes to injury site, or if it is entirely stochastic. Nonetheless, our data clearly show that Tbx5a function is required for cardiac regeneration in zebrafish.

In summary, we demonstrated that a gene trap vector employing lox66/lox72 variant loxP sites in conjunction with tissue specific, tamoxifen-inducible Cre recombinase can be used to inactivate gene expression not only in embryonic, but also in adult zebrafish. We recovered the *tbx5a*^*tpl58*^ mutant from a gene trap screen, which is a random forward genetic approach. However, several recent publications clearly establish the feasibility of integrating transgenes, including fluorescent reporters and a Cre-inducible gene trap cassette, into double strand breaks induced by targeted nucleases [6,50,51,52,53,54]. Non-repetitive nature of lox66/72 sites, in contrast to repetitive nature of the FLeX system, makes our gene trap especially suitable for one-step generation of conditional mutants using reverse genetic approaches.

## Materials and methods

### Animal experiments

All experiments described in this manuscript were approved by Temple University's Institutional Animal Care and Use Committee (IACUC). Anesthesia was performed by immersion into fish water containing tricaine (MS-222, Sigma-Aldrich). Euthanasia was performed either by tricaine overdose or by rapid chilling.

### Gene trap mutagenesis

Plasmids containing gene trap constructs GBT-S1 and GBT-S8 were purified using the Qiagen miniprep protocol. Plasmid DNA was co-injected with Tol2 mRNA into 1-cell wildtype zebrafish embryos as described [55]. At 3 dpf, embryos were screened for high levels of BFP fluorescence using Zeiss Axioscope, and positive embryos were raised to adulthood. Adult F0 founders were crossed to *tg*(*UAS*:*mRFP*)*tpl2* reporter line [19,30]. The F1 progeny was screened for mRFP and BFP expression at 1 and 3 dpf. Adult F1 fish were again outcrossed to establish gene trap lines and to obtain embryos for molecular identification of gene trap events.

Sequences of conditional gene trap vectors have been submitted to GenBank and are available under accession numbers MH450095 (GBT-S1) and MH450096 (GBT-S8).

### Identification of insertionally mutated genes

For inverse PCR, genomic DNA was prepared from batches of 20 RFP-positive and 20 RFP negative embryos, collected at 3–5 dpf. Genomic DNA was then digested with NlaIII, TaqI, NheI/SpeI/XbaI/XmaJI or BamHI/BclI/BglII, then diluted and ligated overnight as described in [56]. Nested inverse PCR reactions were performed with primers Tol2-F8 and S1/5’No1, then Tol2-F10 and S1/3’No2a for the 5’ end of the gene trap, and primers Tol2-R4 and S1/3’No3, then Tol2-R5 then S1/3’No4 for the 3’ end of the gene trap (see **S1 Table** for primer sequences). Bands unique to RFP-positive batches were excised from agarose gel and sequenced with appropriate Tol2 primer (Tol2-F10 for 5’ end, Tol2-R5 for 3’ end). Sequences of PCR fragments were used to perform BLAST searches in NCBI, UCSC and Ensembl databases.

Based on sequencing results, we designed primers in the adjacent genomic DNA and performed PCRs on additional batches of 20 embryos to confirm presence of a particular integration in RFP-positive and but not in RFP-negative embryos.

To further confirm gene trap events, RT PCR was performed using reverse primers in Gal4 and forward primers in an exon 5’ to gene trap integration, on batches of 10–20 RFP-positive and RFP-negative embryos, as described previously [19]. Bands were excised from an agarose gel and sequenced.

### Quantitative RT-PCR

Gene trap heterozygotes were incrossed and embryos with no/high RFP expression were sorted at 1–3 dpf. At 5 dpf, DNA and RNA was prepared from single embryos using the Trizol reagent as previously described [19]. Three-primer PCR (two genomic primers and one gene trap-specific primer) was performed on genomic DNA to identify embryos homozygous for either wild type or the gene trap allele. mRNA from at 3–5 homozygous embryos was pooled, and cDNA was synthesized using Invitrogen Superscript II cDNA synthesis kit. Quantitative PCR was performed in triplicates using Roche LightCycler 480 SYBR Green I kit, with beta-Actin as the reference mRNA. Results of the qPCR were recorded and analyzed using LightCycler 480 software.

### Ventricular resection

Three months old fish were anesthetized in 0.9 mM tricaine (MS222) solution. After exactly 3 min fish were removed from tricaine solution, placed on a wet sponge ventral side up, and ventricular resection was performed as described by Poss and colleagues [57]. A small incision in the area of the heart was made using a pair of small fine scissors. Pericardial sac was opened with fine forceps. About 20% of ventricular apex was carefully removed with small scissors. Upon injury fish was transferred to a recovery tank that was filled with fresh fish system water and had an additional air supply. Recovering fish were maintained at low density (1–5 fish in a 2L tank) on the main water system. All fish were sacrificed at 30 dpa by tricaine overdose or chilling in ice water.

### Histology

Hearts were fixed in 4% PFA, washed in 1x PBS, dehydrated and embedded in paraffin. Eight nm heart sections were made using Leitz 1512 Rotary Microtome. To stain nuclei of the cells, sections were incubated in standard DAPI solution for 10 min, and mounted with water-based mounting media. To detect collagen deposition, sections were rehydrated, stained using Picro-Mallory procedure [58], dehydrated and covered with Vectashield mounting medium.

### Reversion of *tbx5a*^*tpl58*^ gene trap allele using Flp^o^ mRNA

The plasmid coding for Flp^o^ recombinase was a kind gift of Dr. Philippe Soriano [59]. Recombinase coding sequence was cloned into the pT3TS vector [60] to generate pDC50. *In vitro* transcription was performed as described for pT3TS-Tol2 [55]. 75pg of Flp^o^ mRNA was injected into the yolks of embryos obtained from *tbx5a*^*tpl58/+*^, *Tg*(*UAS*:*mRFP*)*tpl2* fish. F0 embryos were screened for mosaic mRFP expression and raised. Mosaic adults were outcrossed to *Tg*(*UAS*:*mRFP*)*tpl2* homozygotes, and F1 (R1) embryos were screened for mRFP expression. The batch that showed the lowest number of mRFP-positive embryos (13%) as compared to the number of mRFP-negative siblings (87%) was used for genotyping. Successful recombination events in F1 embryos and adults were detected via three-primer PCR with tbx5a-F1, tbx5a-R1 and zpa-F2, and confirmed by sequencing. Sequence of pT3TS-Flp^o^ plasmid will be deposited in GenBank.

### Re-trapping of *tbx5a*^*tpl58R*^ locus

Cre mRNA was *in vitro* transcribed and prepared as described previously [17,19] was injected into one-cell embryos from *tbx5a*^*tpl58R/+*^*; UAS*:*mRFP* heterozygous outcross. Embryos were scored for mosaic mRFP expression in the hearts, pectoral fins and dorsal retina (data not shown).

For temporally-controlled re-trapping, *tbx5a*^*tpl58R/+*^*; UAS*:*mRFP* heterozygotes were crossed to the *Ubi*:*CreERT2* ubiquitous CreERT2 driver line [48]. At 2 dpf, embryos were transferred into 0.5 μM 4-hydroxytamoxifen (4-HT hereafter) solution and incubated in the dark for 24 h. At 5 dpf, DNA was prepared from embryos and PCR was performed using primer pairs tbx5aEx1-F1/Gal4-R1 (5’ end) and zpA-F2/eGBYFP-R (3’ end) (**S1 Table**). Obtained bands were excised from agarose gel, purified and sequenced.

For conditional re-trapping in larvae, *tbx5a*^*tpl58R/+*^*; UAS*:*mRFP* heterozygotes were crossed to *Tg(tnnt2a*:*CreERT2*, *crygc*:*mRFP)tpl48*. At 2 dpf, embryos were transferred into embryo water containing 0.5 μM 4-hydroxytamoxifen and incubated in the dark for 24 h. At 3 dpf, embryos were scored for heart-specific mRFP expression.

To re- trap *tbx5a*^*tpl58R*^ locus specifically in the adult heart, 2.5 months old *tbx5a*^*tpl58R/+*^*; tnnt2a*:*CreERT2* and *tbx5a*^*tpl58R/tpl58R*^*; tnnt2a*:*CreERT2* fish were incubated in 5 μM 4-HT solution three times for 24 h with one-day recovery period between treatments. One week after the last incubation, hearts were dissected and screened for mRFP expression. Ventricular resections were performed one week after the last incubation in 4-HT.

## Supporting information

S1 Fig Reversion of *tbx5a*^*tpl58*^ mutant phenotypes by inverting the gene trap cassette. **A, B.** Embryos injected with *Flp*^*o*^ mRNA (A) highly reduced mRFP expression (left) and/or recovery of pectoral fins (right) compared to un-injected siblings (B). **C.** Screening of R1 (F1) embryos for a reverted allele *(tbx5a*^*tpl58R*^*)* by three primer PCR. Embryos in the first three lanes were RFP positive and therefore were expected to carry the non-modified allele *(tbx5a*^*tpl58*^*)*. Embryos in lanes 4–16 were RFP negative and therefore were expected to be either wild-type *(tbx5a*^*+*^*)* or positive for the inverted allele *(tbx5a*^*tpl58R*^*)*. **D**. Fish homozygous for the reverted allele have normal hearts, compared to fish heterozygous for the gene trap. **E**. Enlarged atrium and blunt, mis-shapen ventricle comparable to the one shown in **Fig 2** are indicated by red arrows.
(PPTX)

S2 Fig Sequence analysis of the *tbx5a*^*tpl58*^ gene trap locus (A), Flp-reverted *tbx5a*^*tpl58R*^ locus (B), and re-mutation after cross to Tg(*ubb*:*CreERT2*) (C). **A.** Sequencing of PCR fragments obtained using primer pairs tbx5aEx1-F1/Gal4-R1 (left) and zpA-F2/eGBYFP-R (right) on 5 dpf embryos heterozygous for *tbx5a*^*tpl58*^. **B**. Sequencing of PCR fragments obtained using primer pairs tbx5aEx1-F1/zpA-F2 (left) and Gal4-R1/tbx5aGen-R (right) on a tail clip of a stably reverted *tbx5a*^*tpl58R*^ heterozygote. **C.** Sequencing of PCR fragments obtained using primer pairs tbx5aEx1-F1/Gal4-R1 (left) and zpA-F2/eGBYFP-R (right) on 5 dpf embryos heterozygous for *tbx5a*^*tpl58R*^ and Tg(*ubb*:*CreERT2*), incubated in 0.5 mM 4-HT for 24 hours starting at 2 dpf.
(PPTX)

S3 Fig Tamoxifen-dependent and heart-specific *tg(tnnt2*:*CreERT2)tpl48* driver can re-mutate *tbx5a*^*tpl58R*^ cardiomyocytes, which are then retained in adult hearts. Top, diagram of the miniTol2/tnnt2:CreERT2; crygc:mRFP) transgene. Left, diagram of a Cre-inducible mRFP (flox-mRFP) reporter and heart-specific mRFP expression in 4-HT-treated double transgenic fish. Middle panel, Cre-inducible GFP reporter (flox-GFP) and heart-specific GFP expression in double transgenic fish treated with 4-HT. Right, re-mutation of the gene trap in the cardiomyocytes at 3 dpf. Right bottom, heart of an adult fish which was treated with 4-HT between 2–3 dpf displays mosaic mRFP expression in the atrium and the ventricle, indicating mosaicism for *tbx5a*^*tpl58*^ gene trap re-mutation.
(PPTX)

S1 Table Primer sequences. Sequences of primers mentioned in the text of the manuscript.
(DOCX)
