# Supplementary material for: Analysis of a conditional gene trap reveals that tbx5a is required for heart regeneration in zebrafish
Source: PLoS One. 2018 Jun 22;13(6):e0197293. doi: 10.1371/journal.pone.0197293 (PMC6014646; doi:10.1371/journal.pone.0197293)
Supplement: S1 Fig — A, B. Embryos injected with Flpo mRNA (A) highly reduced mRFP expression (left) and/or recovery of pectoral fins (right) compared to un-injected siblings (B). C. Screening of R1 (F1) embryos for a reverted allele (tbx5atpl58R) by three primer PCR. Embryos in the first three lanes were RFP positive and therefore were expected to carry the non-modified allele (tbx5atpl58). Embryos in lanes 4–16 were RFP negative and therefore were expected to be either wild-type (tbx5a+) or positive for the inverted allele (tbx5atpl58R). D. Fish homozygous for the reverted allele have normal hearts, compared to fish heterozygous for the gene trap. E. Enlarged atrium and blunt, mis-shapen ventricle comparable to the one shown in Fig 2 are indicated by red arrows. (PPTX) [file pone.0197293.s001.pptx]

## Slide 1
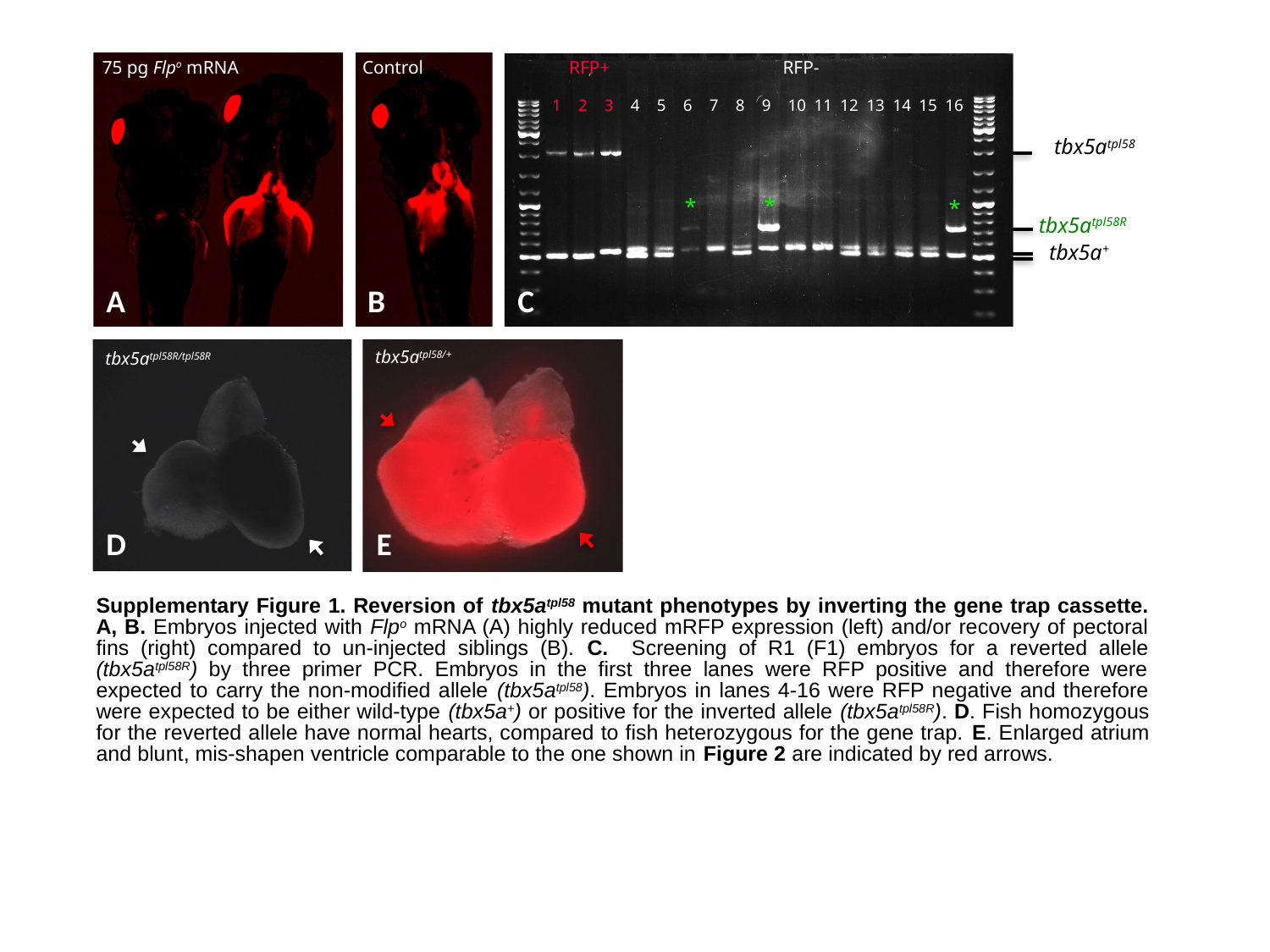

75 pg Flpo mRNA
Control
RFP+
RFP-
1
2
3
4
5
6
7
8
9
10
11
12
13
14
15
16
tbx5atpl58
*
*
*
tbx5atpl58R
tbx5a+
A
B
C
tbx5atpl58/+
tbx5atpl58R/tpl58R
D
E
Supplementary Figure 1. Reversion of tbx5atpl58 mutant phenotypes by inverting the gene trap cassette. A, B. Embryos injected with Flpo mRNA (A) highly reduced mRFP expression (left) and/or recovery of pectoral fins (right) compared to un-injected siblings (B). C. Screening of R1 (F1) embryos for a reverted allele (tbx5atpl58R) by three primer PCR. Embryos in the first three lanes were RFP positive and therefore were expected to carry the non-modified allele (tbx5atpl58). Embryos in lanes 4-16 were RFP negative and therefore were expected to be either wild-type (tbx5a+) or positive for the inverted allele (tbx5atpl58R). D. Fish homozygous for the reverted allele have normal hearts, compared to fish heterozygous for the gene trap. E. Enlarged atrium and blunt, mis-shapen ventricle comparable to the one shown in Figure 2 are indicated by red arrows.
