# Supplementary material for: Analysis of a conditional gene trap reveals that tbx5a is required for heart regeneration in zebrafish
Source: PLoS One. 2018 Jun 22;13(6):e0197293. doi: 10.1371/journal.pone.0197293 (PMC6014646; doi:10.1371/journal.pone.0197293)
Supplement: S2 Fig — A. Sequencing of PCR fragments obtained using primer pairs tbx5aEx1-F1/Gal4-R1 (left) and zpA-F2/eGBYFP-R (right) on 5 dpf embryos heterozygous for tbx5atpl58. B. Sequencing of PCR fragments obtained using primer pairs tbx5aEx1-F1/zpA-F2 (left) and Gal4-R1/tbx5aGen-R (right) on a tail clip of a stably reverted tbx5atpl58R heterozygote. C. Sequencing of PCR fragments obtained using primer pairs tbx5aEx1-F1/Gal4-R1 (left) and zpA-F2/eGBYFP-R (right) on 5 dpf embryos heterozygous for tbx5atpl58R and Tg(ubb:CreERT2), incubated in 0.5 mM 4-HT for 24 hours starting at 2 dpf. (PPTX) [file pone.0197293.s002.pptx]

## Slide 1
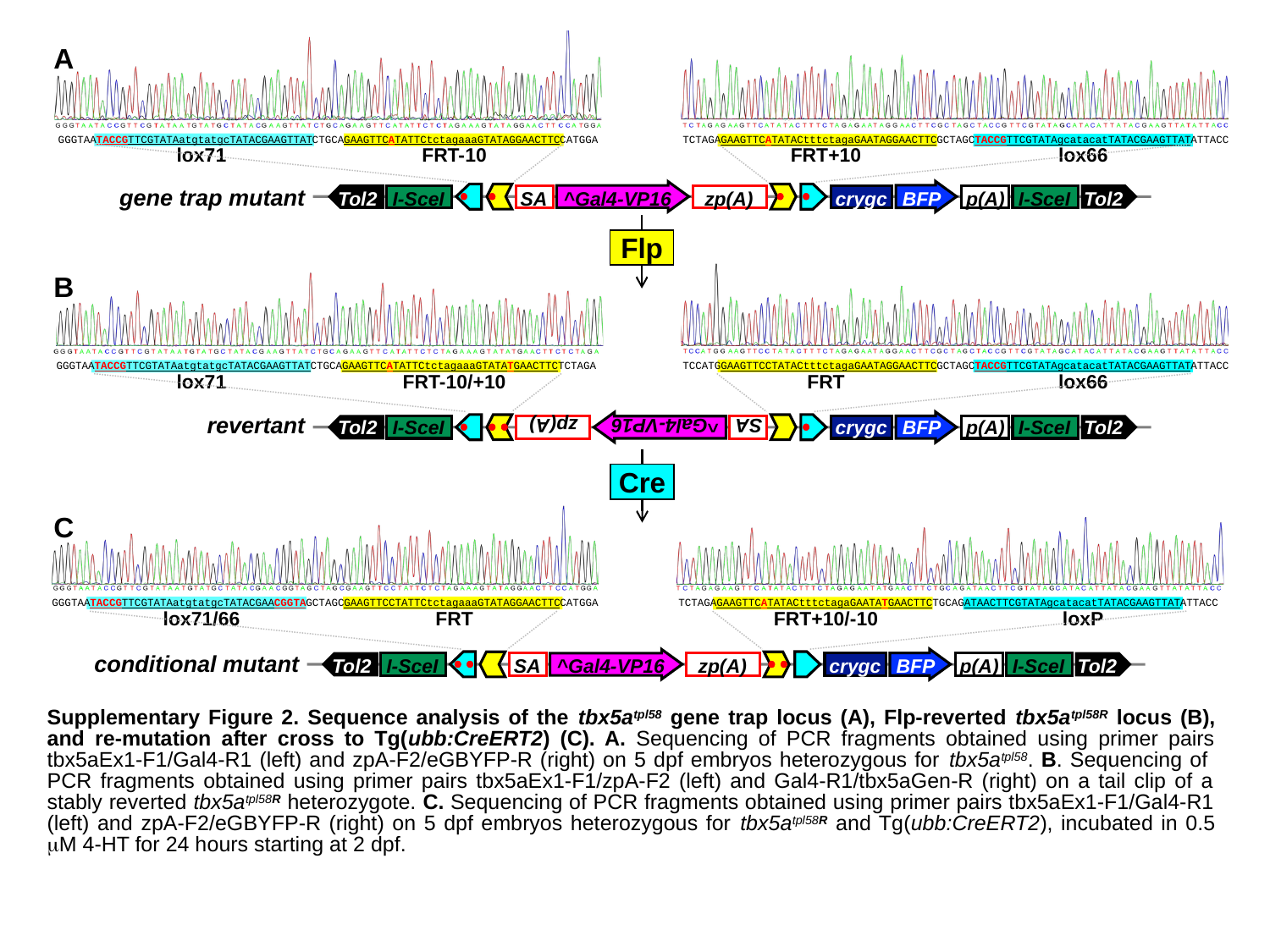

A
GGGTAATACCGTTCGTATAatgtatgcTATACGAAGTTATCTGCAGAAGTTCATATTCtctagaaaGTATAGGAACTTCCATGGA
TCTAGAGAAGTTCATATACtttctagaGAATAGGAACTTCGCTAGCTACCGTTCGTATAgcatacatTATACGAAGTTATATTACC
lox71
FRT-10
FRT+10
lox66
 gene trap mutant
^Gal4-VP16
Tol2
I-SceI
SA
zp(A)
crygc
BFP
p(A)
I-SceI
Tol2
Flp
Tol2
B
TCCATGGAAGTTCCTATACtttctagaGAATAGGAACTTCGCTAGCTACCGTTCGTATAgcatacatTATACGAAGTTATATTACC
GGGTAATACCGTTCGTATAatgtatgcTATACGAAGTTATCTGCAGAAGTTCATATTCtctagaaaGTATATGAACTTCTCTAGA
lox71
FRT-10/+10
FRT
lox66
revertant
^Gal4-VP16
Tol2
I-SceI
zp(A)
SA
crygc
BFP
p(A)
I-SceI
Tol2
Cre
C
GGGTAATACCGTTCGTATAatgtatgcTATACGAACGGTAGCTAGCGAAGTTCCTATTCtctagaaaGTATAGGAACTTCCATGGA
TCTAGAGAAGTTCATATACtttctagaGAATATGAACTTCTGCAGATAACTTCGTATAgcatacatTATACGAAGTTATATTACC
lox71/66
FRT
FRT+10/-10
loxP
conditional mutant
^Gal4-VP16
Tol2
I-SceI
SA
zp(A)
crygc
BFP
p(A)
I-SceI
Tol2
Supplementary Figure 2. Sequence analysis of the tbx5atpl58 gene trap locus (A), Flp-reverted tbx5atpl58R locus (B), and re-mutation after cross to Tg(ubb:CreERT2) (C). A. Sequencing of PCR fragments obtained using primer pairs tbx5aEx1-F1/Gal4-R1 (left) and zpA-F2/eGBYFP-R (right) on 5 dpf embryos heterozygous for tbx5atpl58. B. Sequencing of PCR fragments obtained using primer pairs tbx5aEx1-F1/zpA-F2 (left) and Gal4-R1/tbx5aGen-R (right) on a tail clip of a stably reverted tbx5atpl58R heterozygote. C. Sequencing of PCR fragments obtained using primer pairs tbx5aEx1-F1/Gal4-R1 (left) and zpA-F2/eGBYFP-R (right) on 5 dpf embryos heterozygous for tbx5atpl58R and Tg(ubb:CreERT2), incubated in 0.5 mM 4-HT for 24 hours starting at 2 dpf.
