# Supplementary material for: Analysis of a conditional gene trap reveals that tbx5a is required for heart regeneration in zebrafish
Source: PLoS One. 2018 Jun 22;13(6):e0197293. doi: 10.1371/journal.pone.0197293 (PMC6014646; doi:10.1371/journal.pone.0197293)
Supplement: S3 Fig — Top, diagram of the miniTol2/tnnt2:CreERT2; crygc:mRFP) transgene. Left, diagram of a Cre-inducible mRFP (flox-mRFP) reporter and heart-specific mRFP expression in 4-HT-treated double transgenic fish. Middle panel, Cre-inducible GFP reporter (flox-GFP) and heart-specific GFP expression in double transgenic fish treated with 4-HT. Right, re-mutation of the gene trap in the cardiomyocytes at 3 dpf. Right bottom, heart of an adult fish which was treated with 4-HT between 2–3 dpf displays mosaic mRFP expression in the atrium and the ventricle, indicating mosaicism for tbx5atpl58 gene trap re-mutation. (PPTX) [file pone.0197293.s003.pptx]

## Slide 1
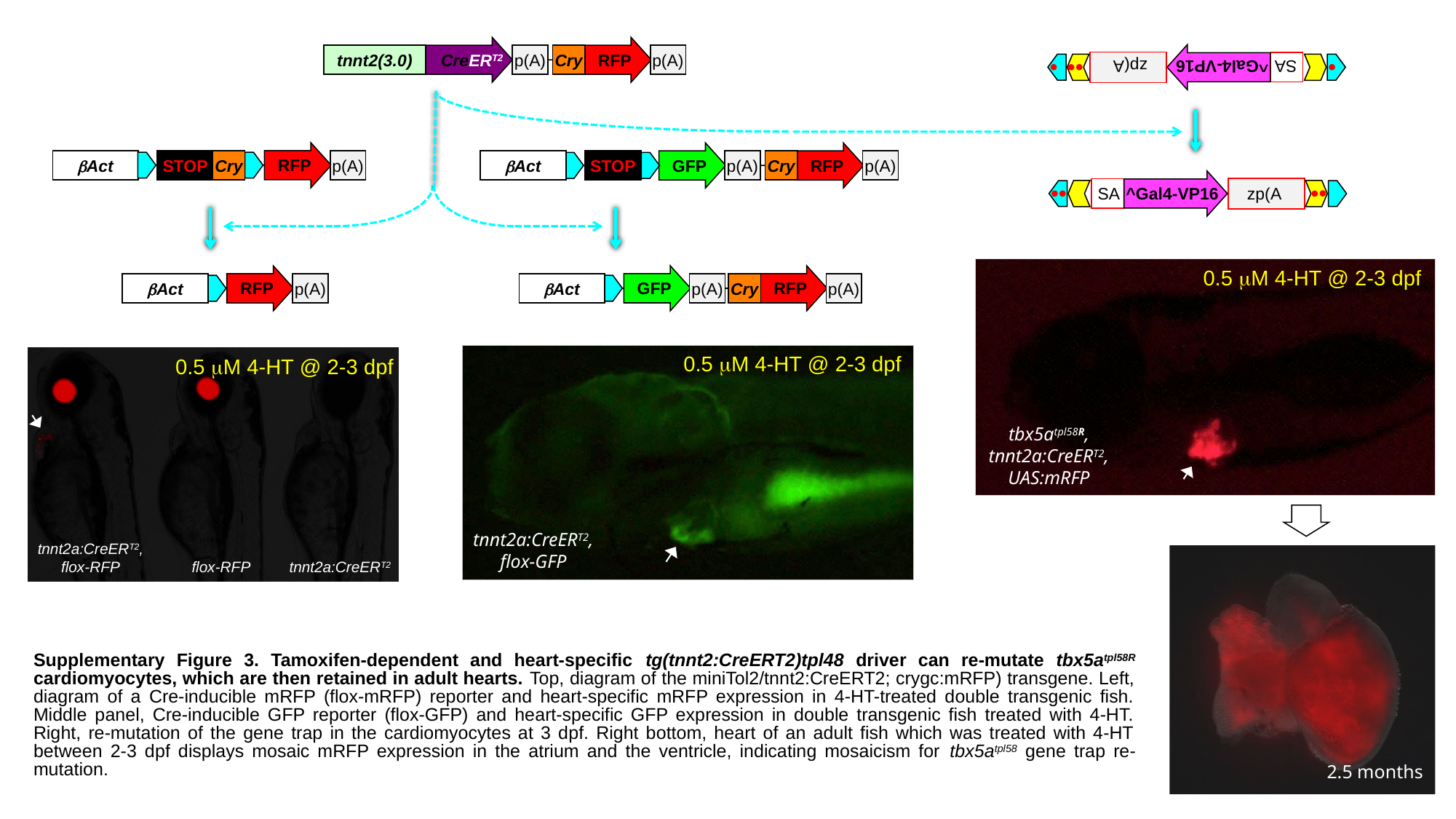

CreERT2
RFP
tnnt2(3.0)
p(A)
Cry
p(A)
zp(A
^Gal4-VP16
SA
RFP
bAct
STOP
Cry
p(A)
RFP
Cry
p(A)
GFP
bAct
STOP
p(A)
SA
^Gal4-VP16
zp(A
0.5 mM 4-HT @ 2-3 dpf
RFP
Cry
p(A)
GFP
RFP
bAct
p(A)
bAct
p(A)
0.5 mM 4-HT @ 2-3 dpf
tnnt2a:CreERT2,
flox-RFP
flox-RFP
tnnt2a:CreERT2
0.5 mM 4-HT @ 2-3 dpf
tbx5atpl58R,
tnnt2a:CreERT2,
UAS:mRFP
tnnt2a:CreERT2,
flox-GFP
Supplementary Figure 3. Tamoxifen-dependent and heart-specific tg(tnnt2:CreERT2)tpl48 driver can re-mutate tbx5atpl58R cardiomyocytes, which are then retained in adult hearts. Top, diagram of the miniTol2/tnnt2:CreERT2; crygc:mRFP) transgene. Left, diagram of a Cre-inducible mRFP (flox-mRFP) reporter and heart-specific mRFP expression in 4-HT-treated double transgenic fish. Middle panel, Cre-inducible GFP reporter (flox-GFP) and heart-specific GFP expression in double transgenic fish treated with 4-HT. Right, re-mutation of the gene trap in the cardiomyocytes at 3 dpf. Right bottom, heart of an adult fish which was treated with 4-HT between 2-3 dpf displays mosaic mRFP expression in the atrium and the ventricle, indicating mosaicism for tbx5atpl58 gene trap re-mutation.
2.5 months
a
