# Supplementary material for: Analysis of a conditional gene trap reveals that tbx5a is required for heart regeneration in zebrafish
Source: PLoS One. 2018 Jun 22;13(6):e0197293. doi: 10.1371/journal.pone.0197293 (PMC6014646; doi:10.1371/journal.pone.0197293)
Supplement: S1 Table — Sequences of primers mentioned in the text of the manuscript. (DOCX) [file pone.0197293.s004.docx]

**Supplementary Table 1. Primer sequences**. Sequences of primers mentioned in the text of the manuscript.

| **Primer Name** | **Sequence 5’ to 3’** |
| --- | --- |
| Tol2-F8 | CTCAAGTAAGATTCTAGCCAGATAC |
| Tol2-F10 | CCCTAAGTACTTGTACTTTCACTTG |
| Tol2-F11 | CCCTTGCTATTACCAAACCAATTGA |
| Tol2-F13 | GTACTTATTTTTTGGAGATCACTTC |
| Tol2-R4 | ATAATACTTAAGTACAGTAATCAAG |
| Tol2-R5 | TAATCAAGTAAAATTACTCAAGTAC |
| Gal4-R1 | TAGCGACACTCCCAGTTGTTCTTCA |
| Gal4-R3 | TAAGTCGGCAAATATCGCATGCTTG |
| Gal4-R4 | GCATGCTTGTTCGATAGAAGACAGT |
| eGBYFP-R | CCGTTTACGTCGCCGTCCAGCTCGA |
| S1/5’No1 | CTAGAGATTCTTGTTTAAGCTTAGG |
| S1/5’No2A | TCTCTAGAAAGTATAGGAACTTCCA |
| S1/3’No3 | TCAGCCATACCACATTTGTAGAGGT |
| S1/3’No4 | CACATTTGTAGAGGTTTTACTTGCT |
| zbAct-F2 | CATATGAAGGTAGCTAACTGCAA |
| tbx5aEx1-F1 | ATCCCCGTCATCGCAGACTACATACATT |
| tbx5a-R1 | CATATGAAGGTAGCTAACTGCAA |
| tbx5a-Gen:R | TTATCAAGTTTAGCTTACCTCTGGACAC |
